# 24-h Movement Guidelines and Substance Use among Adolescents: A School-Based Cross-Sectional Study

**DOI:** 10.3390/ijerph18063309

**Published:** 2021-03-23

**Authors:** Hugues Sampasa-Kanyinga, Ian Colman, Gary S. Goldfield, Ian Janssen, JianLi Wang, Hayley A. Hamilton, Jean-Philippe Chaput

**Affiliations:** 1 School of Epidemiology and Public Health, University of Ottawa, Ottawa, ON K1N 6N5, Canada; icolman@uottawa.ca (I.C.); ggoldfield@cheo.on.ca (G.S.G.); JianLi.Wang@theroyal.ca (J.W.); jpchaput@cheo.on.ca (J.-P.C.); 2 Healthy Active Living and Obesity Research Group, Children’s Hospital of Eastern Ontario Research Institute, Ottawa, ON K1H 8L1, Canada; 3 Centre for Fertility and Health, Norwegian Institute of Public Health, P.O. Box 222 Skøyen, N-0213 Oslo, Norway; 4 School of Kinesiology and Health Studies, Queen’s University, Kingston, ON K7L 3N6, Canada; ian.janssen@queensu.ca; 5 Institute of Mental Health Research, University of Ottawa, Ottawa, ON K1N 6N5, Canada; 6 Institute for Mental Health Policy Research, Centre for Addiction and Mental Health, Toronto, ON M5S 2S1, Canada; hayley.hamilton@camh.ca; 7 Dalla Lana School of Public Health, University of Toronto, Toronto, ON K1N 6N5, Canada

**Keywords:** screen time, sleep duration, physical activity, alcohol consumption, cigarette smoking, cannabis use, teenagers

## Abstract

Children and youth are recommended to achieve at least 60 min/day of moderate-to-vigorous physical activity, no more than 2 h/day of recreational screen time, and a sleep duration of 9–11 h/night for 11–13-year-olds or 8–10 h/night for 14–17-year-olds. Meeting the physical activity, screen time, and sleep duration recommendations have previously been associated with substance use among adolescents. However, previous research has mainly examined these factors individually rather than looking at how these indicators could concurrently relate to substance use in this age group. Therefore, this study examined the associations between meeting the 24-h movement guidelines for screen time, sleep duration, and physical activity (independent variables) with substance use outcomes including alcohol consumption, cannabis use, and cigarette smoking (dependent variables) among adolescents. Self-reported data from a cross-sectional and representative sample of 10,236 students (mean age = 15.1 years) in Ontario, Canada were analyzed. Logistic regression models stratified by gender were adjusted for potential confounders. Combinations of 24-h movement guidelines was differentially associated with substance use in boys and girls. Overall, findings showed that meeting 24-h movement guidelines is associated with lower odds of alcohol consumption, cannabis use, and cigarette smoking differentially with type of recommendation met and gender. Given that the associations between 24-h movement guidelines and substance use differ between boys and girls, future efforts should take this into consideration.

## 1. Introduction

Substance use in adolescence is a major public health problem around the world. It is often initiated in adolescence and continues into adulthood [1]. Tobacco, alcohol, and cannabis are among the substances commonly misused by adolescents [1]. Smoking is a preventable cause of illness and death [2]. It is associated with a higher risk of several cancers, respiratory diseases, heart disease, and strokes. A recent study using World Health Organization data on tobacco use among adolescents aged 13–15 years documented a prevalence of tobacco use of 19.3% [3]. Alcohol is the most commonly consumed substance by adolescents [4], and it has several adverse health effects, including loss of coordination, memory loss, and liver damage. It can also affect behavior and decision-making. Alcohol poisoning can cause adolescents to engage in other risky behaviors, such as impaired driving, drug use, and risky sexual behavior [5]. Alcohol consumption also increases the risk of unintentional injuries, which are the leading cause of death among adolescents [6]. The prevalence of past-year alcohol use among Canadian students in grades 7 through 12 for 2016–2017 was 44% [7]. Cannabis use impairs decision-making skills and judgment and can lead to negative results. It can also be addictive and is associated with harms such as withdrawal symptoms, depression, overdose, and even death [8]. Research indicated a significant increase in the use of cannabis among adolescents in 2017, as their perception of risk decreased [9]. An estimated one in 5 (22%) students in grades 7 through 12 across Ontario, Canada used cannabis in 2019 [10]. Identifying important factors that could reduce the risk of substance use is important to inform prevention efforts.

Some factors that have received greater attention over the past decades are physical activity, screen time, and sleep duration. In general, active living and sufficient sleep duration have many health benefits and contribute substantially to the quality of life across the lifespan [11,12,13,14]. More specifically, physical activity, screen time, and sleep duration have been associated with the use of a variety of substances such as cigarette, alcohol, and cannabis [15,16]. For example, Ströhle et al. [17] found that adolescents who exercised daily or several times per week had a reduced prevalence of having any substance use disorder in the past 12 months compared with adolescents who exercised ≤1 time per month. Studies have also shown that excessive screen time is associated with greater risk of substance use among adolescents [18,19]. Pasch et al. [20] found that short sleep duration prospectively predicted adolescents’ cigarette, alcohol, and marijuana use in a sample of 704 US adolescents. Short sleep duration has also been associated with alcohol consumption and cannabis use in adolescents [21].

However, these lifestyle behaviors (i.e., physical activity, screen time, and sleep duration) have often been examined in isolation, ignoring how they interact with each other. The Canadian 24-h Movement Guidelines for Children and Youth account for the interaction and codependence between physical activity, sedentary behavior, and sleep [22]. The guidelines contain specific recommendations on the time that 5- to 17-year-olds should spend in moderate-to-vigorous physical activity (MVPA; at least 60 min per day), no more than 2 h of recreational screen time per day, and a sleep duration of 9 to 11 h per night for 5- to 13-year-olds, and 8 to 10 h/night for 14- to 17-year-olds to support health benefits [22]. It is estimated that 3 to 10% of adolescents meet all three recommendations [23,24,25,26,27,28]. This is troubling because children and youth who meet all 3 recommendations are more likely to have better cognitive function [25], less impulsivity [29], lower odds of obesity [27], better dietary patterns [30], enhanced quality of life [31], and fewer depressive symptoms [23,24] than children who meet none of the recommendations.

Research has indicated that boys are more likely than girls to engage in some types of substance use, and previous studies have found important gender differences in the relationship between physical activity and alcohol consumption, cannabis use, and cigarette smoking [32]. Recently, Knell et al. [23] documented that compliance to all three recommendations was related to greater odds of smoking cigarettes and lower odds of cannabis use among US male adolescents; whereas among females, it was associated with lower odds of alcohol consumption. Contrary to our study, Knell et al. did not examine the intermediate combinations of meeting the physical activity, screen time, and sleep duration recommendations.

Most research studies have mainly examined the associations between physical activity, screen time, and sleep duration and substance use among adolescents individually rather than looking at how these indicators could concurrently relate to substance use in this age group. However, the relationships between different combinations of movement behaviors and substance use among adolescents is largely unknown. It is thus unknown whether adhering to all or certain combinations of guideline recommendations is associated with a better substance use profile among adolescents. Gaining such knowledge is important because substance use, such as alcohol consumption, cannabis use, and cigarette smoking are of particular concern among adolescents [4,33], they account for a substantial proportion of the global burden of disease in terms of disability-adjusted life years [34], and they cluster together with other unhealthy behaviors (e.g., lack of physical activity, unhealthy eating, etc.) within individuals [35,36,37,38].

To address this knowledge gap, and to inform public health efforts against adolescent substance use, the present study examined the associations between adherence to the recommendations in the Canadian 24-h Movement Guidelines and substance use among adolescents. It was hypothesized that adherence to the Canadian 24-h Movement Guidelines would be associated with lower substance use among adolescents, particularly among boys and younger adolescents because they tend to meet the guidelines more frequently than girls and older adolescents.

## 2. Materials and Methods

Data from the 2017 cycle of the Ontario Student Drug Use and Health Survey (OSDUHS, Canada) were analyzed. The OSDUHS is a biennial cross-sectional survey of Ontario students enrolled in grades 7 through 12 (aged 11 to 20 years or over) in publicly funded schools, representing nearly 93% of the Ontario adolescent population [4]. The survey uses a complex design. Within a selected school, classes are randomly selected within grades and all students in selected classes are asked to complete a self-administered paper-and-pencil questionnaire during a regularly scheduled classroom period. In total, 11,435 students in grades 7–12 from 764 classes across 214 schools completed the 2017 cycle of the survey. Response rates among schools, classes, and students were 61%, 94%, and 61%, respectively. Absenteeism accounted for 11% of the non-participation rate and parental or student refusal or unreturned consent forms accounted for 26%. All subjects gave their signed assent in addition to parentally signed consent for those aged under 18 years before they participated in the study. The study was conducted in accordance with the Declaration of Helsinki, and the protocol was approved by the Research Ethics Boards at the Centre for Addiction and Mental Health and York University, as well as 31 school board research review committees. Detailed information on the OSDUHS are provided elsewhere [4].

### 2.1. Measures

#### 2.1.1. Independent Variables

The measures for individual movement behaviors were obtained from the Centre for Disease Control (CDC)’s Youth Risk Behavior Survey (YRBS) [39].

Physical activity was measured using an item that asked students how many of the last 7 days they were physically active for at least 60 min each day. Students were asked to (1) add up all the time they spent in any types of physical activity that increased their heart rate and made them breathe hard some of the time (such as biking, running, brisk walking, dancing, rollerblading, football, soccer, skateboarding, basketball, and swimming); and (2) to include both school and non-school activities. Response options ranged from 0 to 7 days. For analysis, “7 days” corresponded to students that met the physical activity guideline recommendation [22]. The remaining responses were combined to represent those who did not meet the physical activity recommendation. This self-reported measure has shown good validity in comparison with accelerometry measures among children and adolescents [40].

Screen time was assessed using an item that asked students to indicate how many hours a day, on average, they spend watching TV/movies/videos, texting, playing video/computer games, emailing, or surfing the Internet in their free time. Spending no more than 2 h per day of screen time represented adherence to the screen time recommendation [22]. Not meeting the screen time recommendation represented a screen time of more than 2 h per day. Measures of recreational screen time have also demonstrated good psychometric properties in this age group [41,42].

Sleep duration was assessed using a question that asked on an average school night, how many hours of sleep do students get. A binary variable was created defining adherence to the sleep duration based on the recommended range (9–11 h per night for 11–13-year-olds; 8–10 h per night for 14–17-year-olds, or 7–9 h per night for those aged 18 years or older), contrasting with getting insufficient sleep [14,22]. This self-reported measure of sleep duration has shown good validity and reliability among children and adolescents [43].

#### 2.1.2. Outcome Variables

Similar to the independent variables of physical activity, screen time, and sleep duration, outcome measures were also derived from the CDC’s YRBS [39]. Alcohol use was measured with a question that asked students how often they drink alcohol (liquor, wine, beer, coolers) over the past 12 months. Tobacco use was measured with an item that asked students how often they smoke cigarettes over the past 12 months. Cannabis use was measured with a question that asked students how often they use cannabis (e.g., “marijuana”) over the past 12 months. For all three, responses were dichotomized, contrasting substance use at least once vs. non-use over the past 12 months. Participants who indicated that they had a few puffs for tobacco cigarette or consumed a sip for alcohol were included in the non-use group, because a few puffs of tobacco cigarette and a sip of alcohol are different from regular use of these substances. A sensitivity analysis using a different cut-off (i.e., a high level of substance use) was run to contrast “regular use” vs. “non-regular use.” Specifically, regular substance use was defined as at least 3 to 5 cigarettes smoking/day, at least 2 or 3 times a month of alcohol consumption, and 6 to 9 times of cannabis use in the last 12 months and were contrasted to the respective non-regular substance use.

#### 2.1.3. Covariates

Covariates included age, ethnicity, gender, body mass index (BMI) *z*-score, and subjective socioeconomic status (SES). Age was measured in years, gender determined by being boy or girl, and ethnicity captured different ethnoracial backgrounds, including White, Black, East and South-East Asian, South Asian, and Other. Subjective SES was assessed using an adapted version of the MacArthur Scale of Subjective Social Status [44]. Students self-reported their body weight (kilograms) and height (meters). We calculated BMI (km/m^2^) and converted it into *z*-scores following the World Health Organization’s reference data [45].

### 2.2. Statistical Analysis

Participant characteristics were described via proportions and means. A Pearson’s χ^2^ test adjusted for the complex survey design (for categorical variables) and an adjusted Wald test were used to test (for continuous variables) the statistical differences between boys and girls and between 11- to 14-year-olds and 15- to 20-year-olds. Correlations between substance use and meeting individual movement recommendations were examined using a combination of pairwise Pearson’s and Spearman correlations. The strength of the correlation coefficients was categorized as follows: weak (less than 0.4), moderate (0.4 to 0.7), and strong (more than 0.7) [46]. Subsequent analyses were conducted in boys and girls separately, because a two-way interaction between gender and movement behaviors was statistically significant (*p* < 0.05) for all outcomes. However, for cannabis use only, a two-way interaction between age and movement behaviors was significant, but not three-way interaction between gender, age, and movement behaviors; thus, analyses for cannabis use were also stratified by age groups. Meeting individual guideline recommendations was binary coded as 0 (not meeting the guideline) or 1 (meeting the guideline). A new variable was constructed to represent different combinations of the three guideline recommendations, thus generating eight categories (i.e., meeting none, meeting the physical activity recommendation only, meeting the screen time recommendation only, meeting the sleep duration recommendation only, meeting the physical activity and screen time recommendations only, meeting the physical activity and sleep duration recommendations only, meeting the screen time and sleep duration recommendations only, meeting all three recommendations) where meeting none of the guidelines was treated as the reference category.

The association between meeting individual and different combinations of movement behavior guidelines (independent variables) and substance use outcomes of alcohol consumption, tobacco smoking, and cannabis use (dependent variables) were examined using logistic regression analyses. The regression models controlled for ethnicity, gender, BMI *z*-score, and subjective SES as potential confounders. A total of 10,236 participants out of 11,435 were retained for our analyses, because they had complete information on all variables of interest. Compared to included participant, those who were excluded (due to missing data) were more likely to be aged 11-to-14-years (58.6% vs. 37.7%), less likely to be of a White ethnicity (48.0% vs. 56.2%), had higher BMI *z*-scores, and were more likely to meet the sleep duration recommendation (44.8% vs. 33.8%) (Table S1). Given that the OSDUHS uses a two-stage (school, class) stratified (region and school type) cluster sample design, all analyses were adjusted for this complex sample design using Taylor series linearization methods in Stata 16.0 (Stata Corporation, College Station, TX, USA).

## 3. Results

Table 1 outlines descriptive characteristics of the sample. Just over two-thirds were 15-to-20-year-olds, almost one-half were girls, and 56.3% identified themselves as White. Overall, 39% met none of the recommendations, 7.7% met the physical activity recommendation only, 13.8% met the screen time recommendation only, and 15.4% met the sleep duration recommendation only. Only 5% of participants met all three recommendations. The proportion of boys and girls meeting the sleep duration recommendation only was not significantly different (*p* = 0.093). Boys were more likely than girls to meet the physical activity recommendation only (*p* < 0.001), whereas girls were more likely than boys to meet the screen time recommendation only (*p* < 0.001). Boys were more likely than girls to meet all three recommendations (*p* < 0.001). Boys were also more likely than girls to report smoking tobacco cigarette (*p* = 0.037). The prevalence of adherence to the screen time, sleep duration, and physical activity recommendations was greater for 11 to 14-year-olds than for 15-to-20-year-old counterparts (*p* < 0.001). Conversely, 15-to-20-year-olds were more likely than 11-to-14-year-olds to report smoking tobacco cigarette, consuming alcohol, and using cannabis (*p* < 0.001) (Table 1).

Correlation coefficients among substance use and meeting individual guideline recommendations are presented in Table 2. Alcohol consumption and cigarette smoking were moderately associated with cannabis use. Overall, the correlation between 24-h movement guidelines and substance use were statistically significant but weak. Adherence to the physical activity recommendation was positively associated with adherence to the sleep duration and screen time recommendations. Conversely, it was negatively associated with alcohol consumption, cannabis use, and cigarette smoking. Adherence to the screen time recommendation was positively associated with adherence to the sleep duration recommendation. However, it was negatively associated with alcohol consumption and cannabis use. Adherence to the sleep duration recommendation was negatively associated with smoking tobacco cigarette, alcohol consumption, and cannabis use.

As shown in Table 3, after adjusting for important covariates, results from logistic regression analyses showed that among boys, adherence to the physical activity recommendation only was associated with lower odds of smoking tobacco cigarette (OR = 0.31; 95% CI: 0.14–0.68), adherence to the sleep duration recommendation only was associated with lower odds of alcohol consumption (OR = 0.47; 95% CI: 0.31–0.72), and meeting both the physical activity and screen time recommendations was associated with a lower odds of cannabis use (OR = 0.55; 95% CI: 0.32–0.96). Surprisingly, meeting both the physical activity and screen time recommendations was associated with greater odds of alcohol consumption (OR = 1.54; 95% CI: 1.05–2.25). Among girls, meeting all three recommendations was associated with lower odds of cannabis use (OR = 0.35; 95% CI: 0.17–0.72); adherence to the physical activity and sleep duration recommendations only was associated with lower odds of smoking tobacco cigarette (OR = 0.02; 95% CI: 0.00–0.18). Adherence to the screen time recommendation only (OR = 0.68; 95% CI: 0.48–0.96), sleep duration only (OR = 0.53; 95% CI: 0.36–0.78), or both screen time and sleep duration (OR = 0.32; 95% CI: 0.22–0.47) recommendations was associated with lower odds of alcohol consumption. Adherence to the physical activity recommendation only was associated with lower odds of cannabis use (OR = 0.68; 95% CI: 0.47–0.99).

As shown in Table 4, adherence to the sleep duration recommendation only (OR = 0.48; 95% CI: 0.27–0.85) or the screen time and sleep duration recommendations only (OR = 0.35; 95% CI: 0.13–0.91) were associated with lower odds of cannabis use among 11- to-14-year-olds, but not among 15-to-20-year-olds.

Results of sensitivity analyses using more severe levels of substance use as the exposure are outlined in Tables S2 and S3. Overall, they showed similar patterns of association, but with some exceptions and very wide confidence intervals.

## 4. Discussion

This is the first study to examine the association between all possible combinations of screen time, sleep duration, and physical activity with substance use among adolescents. Past research has mainly focused on investigating the associations between a single recommendation of the guidelines and substance use among adolescents [15,16,17,18,19,20,21,32], ignoring how these behaviors may concurrently relate to substance use among adolescents. We observed that different combinations of the guideline recommendations were associated with all substance use differentially between type of recommendation met, type of substance use, and gender, as well as age for cannabis use. Contrary to our hypothesis that adherence to the Canadian 24-h movement guidelines would be associated with reduced substance use among adolescents, particularly among boys and younger adolescents as they are more likely than girls to meet all three recommendations, we observed that meeting all three guideline recommendations was associated with lower odds of cannabis use in girls only. These findings are somewhat surprising because meeting all 3 guideline recommendations should be strongly (lower odds) associated with all the outcomes, and behaviors tend to cluster within individuals. Our analyses may be underpowered to detect small and, in some cases, moderate differences. Future studies with more statistical power are needed to replicate our analyses.

Meeting movement behavior guidelines has been associated with improved indicators of physical, mental, and social health, such as less impulsivity, better self-regulation, cognition, and decision-making, higher social connectedness, and better psychosocial functioning [25,47]. All these outcomes have been indicated to protect adolescents from engaging in risky behaviors such as substance use [48,49]. It is also possible that adolescents who do not use substances have a better self-regulation, and therefore could be more likely to value and lead a healthy lifestyle and meet the guidelines. Regardless, research has shown that unhealthy behaviors (e.g., lack of physical activity, smoking, alcohol, unhealthy eating, etc.) tend to cluster together within individuals [35,36,37,38]. As such, from an intervention standpoint, reducing substance use among adolescents should be integrated within broader health-promoting initiatives.

Our results showed that adherence to the physical activity recommendation was associated with lower odds of smoking tobacco cigarette and cannabis use among boys. Whereas among girls, meeting both the physical activity and sleep duration recommendations was associated with lower odds of smoking tobacco cigarette; and, adherence to the physical activity recommendation only or all three recommendations were associated with lower odds of cannabis use. Our results are in line with previous research suggesting that physical activity may protect adolescents from smoking and cannabis use [50,51]. In a prospective cohort study of over 1300 US adolescents aged 14 to 18 years, Audrain-McGovern and Rodriguez [52] found that the influence of physical activity on the uptake of cigarette smoking largely depends on the types of physical activity youth engage in. They found that physical activity types that are associated with lower odds of smoking include racquet sports, running, and swimming laps, whereas skating, walking, bicycling, sport fighting, and competitive wrestling are positively associated with smoking [52]. In a sample of over 6800 Norwegian adolescents aged 13–19 years, Holmen et al. [53] found that participating in sports that involve lesser endurance, such as fighting sports or body-building was related to daily smoking cigarettes. Findings from the present study do not discriminate types of physical activity, but support that getting 60 min of daily physical activity at moderate to vigorous intensity (MVPA) was associated with lower odds of tobacco and cannabis use, but further research into intensity, duration and type of physical activity is needed. However, the finding that physical activity was not negatively associated with smoking and cannabis use when it was paired with adherence to the sleep duration recommendation among boys suggests that sufficient sleep duration could explain this relationship. Future research formally testing the mediating role of sufficient sleep on the relationship between physical activity and substance use is needed.

Adherence to the sleep duration recommendation only was associated with lower odds of alcohol consumption among boys. However, adherence to the sleep duration recommendation only, screen time recommendation only, or both was associated with lower odds of alcohol consumption among girls. These findings identify sleep duration as an important correlate of alcohol consumption and are somewhat consistent with previous studies that have found that short sleep duration is associated with the age of onset and total alcohol consumption among adolescents [20,21,54] and adults [55]. Studies have also shown that screen time is associated with greater risk of alcohol consumption and binge drinking among adolescents [18,56]. It is possible that higher screen use increases exposure to alcohol advertising, which in turn may result in more positive beliefs about drinking and desire for alcohol initiation, which are predictive of underage drinking [56,57,58,59,60,61,62]. The observed gender differences in the associations between combinations of movement guidelines and alcohol consumption are interesting and deserve more investigation. It is well-known that adult men are more likely than women to consume alcohol [63,64]; however, current literature is inconsistent for such differences in adolescents [65,66], indicating the gender differences in association with movement behaviors are not simply a reflection of higher alcohol use in one gender over another. Research indicated that gender differences in alcohol use become more apparent around the age of 18 [67].

Surprisingly, meeting both the physical activity and screen time recommendations was associated with greater odds of alcohol consumption. These findings are contrary to our hypothesis that adherence to the 24-h movement guidelines would be negatively associated with substance use among adolescents and deserve further investigation. There is compelling evidence that physical activity, particularly competitive sport, is positively associated with current and future alcohol use among adolescents and young adults [15,68]. Research has also suggested that adolescents who play sports intensively drink more than those who practice sports in moderation [69], and those who participated in group sports drink more than those who participated as individuals [70,71]. However, our survey did not ask about specific activities youth engage in while exercising nor the intensity of such activities. Future research is needed to examine how different types and intensities of physical activity relate to alcohol consumption among adolescents.

Our results showed that meeting both the physical activity and screen time recommendations was associated with lower odds of cannabis use among boys. Whereas among girls, adherence to the physical activity recommendation only or all three recommendations was associated with lower odds of cannabis use. Our results are somewhat consistent with previous studies indicating a negative association between physical activity and cannabis use [15]. For example, Wichstrøm and Wichstrøm [71] found that participation in team sports and endurance sports may reduce later cannabis use. Furthermore, physical activity has also been identified as an adjunctive treatment for cannabis use disorder [72]. Our results suggest that adherence to the 24-h movement guidelines, particularly the physical activity component, may play an important role in the prevention of cannabis use among adolescents. Contrary to Knell et al. [23] who found that meeting all three recommendations was associated with lower odds of cannabis use among boys, the present study documented such an association among girls, but not boys. The observed difference may be explained at least in part by the older age of the US sample (high school students), whereas our study included both middle and high school students.

Results further indicated that adherence to the sleep duration recommendation only or both the sleep duration and screen time recommendations was associated with lower odds of cannabis use among 11- to 14-year-olds but not among 15- to 20-year-olds. Our results are consistent with previous studies that have identified sleep duration as an important correlate of cannabis use among adolescents. For example, in a prospective study of over 800 US adolescents aged 12 to 16 years, Miller et al. [21] found that short sleep duration was associated with increased odds of cannabis use. These findings suggest that compliance to the sleep duration and screen time recommendations have added benefits to protect against cannabis use than compliance to the physical activity recommendation among younger adolescents. Excessive screen time is very common among adolescents [73,74], and it is associated with short sleep duration in this age group [75,76]. Replication studies using a longitudinal design are needed to confirm if adherence to the sleep duration and screen time recommendations could help spare younger adolescents from engaging in cannabis use.

### Strengths and Limitations

Strengths of this study include: (1) The fact that it was based on a large and representative sample of the Ontario school population; (2) an assessment of multiple substance use outcomes, specifically alcohol consumption, cannabis use, and cigarette smoking; (3) a focus on adherence to different combinations of movement behavior recommendations, as previous studies have been limited to adherence to individual recommendations; and (4) the inclusion of a comprehensive set of covariates that statistically controlled for important confounding variables, strengthening the internal validity of the findings. Some limitations of the study should be noted. First, the cross-sectional nature of the data precludes temporality and inference of causality between adherence to the 24-h movement guidelines and substance use. Second, physical activity, screen time, sleep duration, and substance use were based on self-reports, and may be subject to recall or desirability biases. Third, the present study did not measure types of physical activity nor screen time. Research has shown that different types/intensities of physical activity and different types of screen time could be differentially associated with substance use in children and adolescents [77,78]. Fourth, the use of single item measures of alcohol consumption, cannabis use, and cigarette smoking may raise reliability issues. Finally, the survey was restricted to adolescent students enrolled in publicly funded schools, thus excluding by design out-of-scope groups for which substance use is elevated, such as institutionalized youths, truant youth, and homeless/street youth.

## 5. Conclusions

Findings from this study suggest that adherence to the 24-h movement behavior recommendations is differentially associated with lower odds of alcohol consumption, cannabis use, and cigarette smoking among adolescent boys and girls. Overall, meeting all of the 24-h movement behavior guidelines was not associated with substance use in boys, and even in girls, only less cannabis use was. Rather, certain individual or combinations of guidelines were more associated with substance use in boys and girls. No gradient or dose response was documented in our data. More specifically, our study identifies physical activity as an important correlate of reduced cigarette smoking, short sleep duration as an important correlate of alcohol consumption, and both physical activity and sleep duration as important correlates of lower cannabis use among adolescents. Replications studies using a larger sample size, objective measures of 24-h movement behaviors, and prospective data are needed to confirm our findings, explain the observed differences across gender and age, and examine if encouraging students to meet the 24-h movement guidelines could protect against substance use.

## Figures and Tables

**Table 1:** Descriptive characteristics of the study sample.

Characteristics	Total Sample (*n* = 10,236)	Boys (*n* = 4431)	Girls (*n* = 5805)	*p* Value	11-to-14-Year-Olds (*n* = 4871)	15-to-20-Year-Olds (*n* = 5365)	*p* Value
Age (years)							
Mean (SD)	15.1 (1.8)	15.2 (1.7)	15.1 (1.9)		13.2 (0.9)	16.3 (0.9)	
11-to-14-year-olds	37.7	37.1	38.3	0.515			
15-to-20-year-olds	62.3	62.9	61.7				
Gender							
Boys	51.1				49.6	48.4	0.515
Girls	48.8				50.4	51.6	
Ethnic background							
White	56.3	58.6	54.0	0.210	54.9	57.2	0.367
Black	9.8	9.5	10.1		9.3	10.1	
East/South-East Asian	8.8	8.4	9.2		8.3	9.1	
South Asian	7.1	7.2	6.9		8.5	6.2	
Other	18.0	16.3	19.8		18.9	17.5	
Subjective socioeconomic status							
Mean (SD)	6.9 (1.7)	7.0 (1.6)	6.9 (1.8)	0.556	7.1 (1.8)	6.8 (1.6)	<0.001
Body mass index *z*-score							
Mean (SD)	0.3 (1.1)	0.3 (1.0)	0.3 (1.3)	0.555	0.3 (1.3)	0.4 (1.0)	0.003
Combination of meeting movement guideline recommendations							
Meeting none	39.0	34.0	44.2	<0.001	30.5	44.2	<0.001
PA only	7.7	9.7	5.6		9.7	6.5	
ST only	13.8	11.5	16.2		12.1	14.8	
Sleep only	15.4	16.4	14.3		14.8	15.7	
PA and ST only	5.9	7.5	4.4		8.0	4.7	
PA and sleep only	4.6	6.0	3.1		6.5	3.4	
ST and sleep only	8.6	7.6	9.6		11.4	6.9	
Meeting all 3	5.1	7.4	2.7		7.0	4.0	
Cigarette smoking							
No	92.6	91.3	93.9	0.037	98.7	88.9	<0.001
Yes	7.4	8.7	6.1		1.3	11.1	
Alcohol consumption							
No	55.6	55.1	56.0	0.696	81.1	40.1	<0.001
Yes	44.4	44.9	44.0		18.9	59.9	
Cannabis use							
No	77.7	77.2	78.2	0.442	94.8	67.3	<0.001
Yes	22.3	22.8	21.8		5.2	32.7	

Data are shown as column %, unless otherwise indicated. SD: standard deviation.

**Table 2:** Spearman’s correlation coefficients among meeting individual guideline recommendations and substance use among adolescents.

	Physical Activity	Screen Time	Sleep	Smoking	Alcohol	Cannabis
Physical activity	1					
Screen time	0.14 ***	1				
Sleep	0.10 ***	0.13 ***	1			
Smoking	−0.03 ***	0.00	−0.05 ***	1		
Alcohol	−0.04 ***	−0.03 ***	−0.11 ***	0.29 ***	1	
Cannabis	−0.05 ***	−0.04 ***	−0.09 ***	0.45 ***	0.51 ***	1

*** *p* < 0.001.

**Table 3:** Association between different combinations of movement behavior recommendations and substance use by gender.

	Cigarette Smoking		Alcohol Consumption		Cannabis Use	
	Boys (*n* = 4431)	Girls (*n* = 5805)	Boys (*n* = 4431)	Girls (*n* = 5805)	Boys (*n* = 4431)	Girls (*n* = 5805)
	OR (95% CI)	OR (95% CI)	OR (95% CI)	OR (95% CI)	OR (95% CI)	OR (95% CI)
**Unadjusted**						
Meeting none	1	1	1	1	1	1
PA only	**0.28 (0.13–0.60)**	0.84 (0.43–1.64)	0.75 (0.47–1.20)	0.81 (0.58–1.13)	0.87 (0.59–1.28)	**0.68 (0.48–0.98)**
ST only	0.62 (0.35–1.10)	1.05 (0.61–1.81)	0.88 (0.61–1.27)	0.84 (0.60–1.16)	0.83 (0.57–1.22)	0.82 (0.60–1.14)
Sleep only	0.83 (0.46–1.49)	0.78 (0.34–1.77)	**0.61 (0.40–0.93)**	**0.68 (0.51–0.92)**	0.60 (0.35–1.03)	0.84 (0.63–1.12)
PA and ST only	**0.39 (0.19–0.80)**	**0.40 (0.18–0.92)**	1.08 (0.79–1.48)	1.16 (0.74–1.82)	**0.56 (0.34–0.93)**	0.79 (0.33–1.88)
PA and sleep only	0.45 (0.19–1.05)	**0.02 (0.03–0.16)**	**0.47 (0.30–0.75)**	0.63 (0.31–1.32)	**0.56 (0.33–0.95)**	0.42 (0.14–1.21)
ST and sleep only	0.62 (0.25–1.55)	**0.49 (0.25–0.94)**	0.78 (0.49–1.24)	**0.32 (0.22–0.47)**	0.71 (0.42–1.18)	0.51 (0.19–1.33)
Meeting all 3	0.92 (0.36–2.36)	0.31 (0.07–1.42)	0.68 (0.45–1.03)	**0.43 (0.24–0.79)**	0.60 (0.33–1.08)	**0.36 (0.18–0.75)**
**Adjusted**						
Meeting none	1	1	1	1	1	1
PA only	**0.31 (0.14–0.68)**	1.19 (0.58–2.48)	0.96 (0.51–1.78)	1.37 (0.94–1.99)	0.86 (0.59–1.27)	**0.68 (0.47–0.99)**
ST only	0.71 (0.40–1.26)	0.92 (0.54–1.57)	0.95 (0.55–1.65)	**0.68 (0.48–0.96)**	0.88 (0.60–1.31)	0.79 (0.57–1.08)
Sleep only	0.72 (0.41–1.28)	0.62 (0.27–1.46)	**0.47 (0.31–0.72)**	**0.53 (0.36–0.78)**	0.61 (0.37–1.01)	0.79 (0.60–1.05)
PA and ST only	0.49 (0.21–1.15)	0.51 (0.22–1.16)	**1.54 (1.05–2.25)**	1.46 (0.91–2.35)	**0.55 (0.32–0.96)**	0.73 (0.29–1.85)
PA and sleep only	0.80 (0.30–2.17)	**0.02 (0.00–0.18)**	0.70 (0.42–1.18)	0.76 (0.32–1.82)	0.66 (0.38–1.14)	0.38 (0.13–1.17)
ST and sleep only	0.73 (0.30–1.77)	0.59 (0.31–1.10)	0.91 (0.42–1.18)	**0.32 (0.22–0.47)**	0.75 (0.46–1.22)	0.49 (0.19–1.30)
Meeting all 3	1.48 (0.63–3.46)	0.41 (0.08–2.09)	0.82 (0.60–1.38)	0.62 (0.35–1.08)	0.67 (0.39–1.13)	**0.35 (0.17–0.72)**

OR: odds ratio; CI: confidence interval; PA: physical activity; ST: screen time. Models are adjusted for ethnicity, gender, body mass index *z*-score, and subjective socioeconomic status.

**Table 4:** Association between different combinations of movement behavior recommendations and cannabis use by age group.

	11-to-14-Year-Olds (*n* = 4871)	15-to-20-Year-Olds (*n* = 5365)
	OR (95% CI)	OR (95% CI)
**Unadjusted**		
Meeting none	1	1
PA only	0.99 (0.57–1.73)	1.10 (0.73–1.66)
ST only	0.61 (0.29–1.29)	0.89 (0.65–1.24)
Sleep only	**0.46 (0.26–0.83)**	0.81 (0.54–1.22)
PA and ST only	0.72 (0.38–1.35)	0.94 (0.60–1.46)
PA and sleep only	0.66 (0.27–1.60)	0.77 (0.43–1.38)
ST and sleep only	**0.34 (0.13–0.90)**	0.93 (0.51–1.72)
Meeting all 3	**0.34 (0.12–0.95)**	0.88 (0.46–1.67)
**Adjusted**		
Meeting none	1	1
PA only	1.02 (0.58–1.78)	1.07 (0.71–1.61)
ST only	0.62 (0.30–1.30)	0.89 (0.65–1.20)
Sleep only	**0.48 (0.27–0.85)**	0.78 (0.52–1.15)
PA and ST only	0.70 (0.37–1.35)	0.88 (0.53–1.47)
PA and sleep only	0.69 (0.30–1.57)	0.78 (0.46–1.34)
ST and sleep only	**0.35 (0.13–0.91)**	0.94 (0.50–1.77)
Meeting all 3	0.37 (0.12–1.09)	0.85 (0.47–1.55)

OR: odds ratio; CI: confidence interval; PA: physical activity; ST: screen time. Models are adjusted for ethnicity, gender, body mass index *z*-score, and subjective socioeconomic status.

## Data Availability

Our data cannot be made available in the manuscript, the supplemental files or a public repositor due to the Centre for Addiction and Mental Health’s and The Ontario Public and Catholic School Board’s institutional Research Ethics Board agreements y. Readers, however, may contact to request the public data file underlying the findings of this study by contacting the Centre for Addiction and Mental Health at info@camh.ca

## Supplementary Materials

The following are available online at https://www.mdpi.com/1660-4601/18/6/3309/s1. Table S1. Descriptive characteristics of participants who were included vs. those who were excluded from our analyses, Table S2. Results of sensitivity analyses testing the link between different combinations of compliance to the movement behavior recommendations and substance use by gender, Table S3. Results of sensitivity analyses testing the association between combinations of adherence to movement behavior recommendations and cannabis use by age group.
