# Supplementary material for: 24-h Movement Guidelines and Substance Use among Adolescents: A School-Based Cross-Sectional Study
Source: Int J Environ Res Public Health. 2021 Mar 23;18(6):3309. doi: 10.3390/ijerph18063309 (PMC8004679; doi:10.3390/ijerph18063309)
Supplement: Supplementary file 1 [file ijerph-18-03309-s001.pdf]

International Journal of
Environmental Research and Public Health

MDPI

Article

24-h Movement Guidelines and Substance Use among Adolescents: A School-Based Cross-Sectional Study

Hugues Sampasa-Kanyinga [1,2,*], Ian Colman [1,3], Gary S. Goldfield [1,2], Ian Janssen [4], JianLi Wang [1,5], Hayley A. Hamilton [6,7] and Jean-Philippe Chaput [1,2]

Table S1. Descriptive characteristics of participants according to whether they were included or excluded from the statistical analyses.

	Included participants (N = 10,258)	Excluded participants (N= 1,177)	P value
Age (years)			
Mean (95% CI)	15.1 (15.0-15.3)	14.3 (13.5-15.3)	0.036
11-to-14-year-olds	37.7 (34.2–41.3)	58.6 (39.6–75.3)	0.030
15-to-20-year-olds	62.3 (58.7–65.8)	41.4 (24.7–60.4)	
Gender			
Boys	51.1 (47.9-54.3)	55.1 (51.5-58.6)	0.102
Girls	48.9 (45.7-52.1)	44.9 (41.4-48.5)	
Ethnic background			
White	56.2 (51.3–61.0)	48.0 (41.1–54.9)	0.037
Black	10.0 (7.6–13.0)	12.4 (6.7–21.8)	
East/South-East Asian	8.8 (7.5–10.4)	8.3 (5.3–12.8)	
South Asian	7.0 (5.7–8.7)	4.9 (3.0–7.8)	
Other	18.0 (15.7–20.5)	26.4 (20.2–33.7)	
Subjective socioeconomic status			
Mean (95% CI)	6.9 (6.8-7.0)	7.0 (6.6-7.4)	0.862
Body mass index z-score			
Mean (95% CI)	0.3 (0.3-0.4)	0.1 (0.0-0.2)	<0.001
Physical activity			
Not meeting	76.8 (75.3–78.2)	78.7 (76.1–81.0)	0.175
Meeting	23.2 (21.8–24.7)	21.3 (19.0–23.9)	
Screen time			
Not meeting	66.4 (64.2–68.7)	72.8 (66.4–78.3)	0.053
Meeting	33.6 (31.4–35.8)	27.2 (21.7–33.6)	
Sleep duration			
Not meeting	66.2 (64.6–67.8)	55.2 (50.5–59.7)	<0.001
Meeting	33.8 (32.2–35.4)	44.8 (40.3–49.5)	
Tobacco cigarette smoking			
No	92.6 (91.2–93.8)	95.8 (90.9–98.1)	0.135
Yes	7.4 (6.2–8.8)	4.2 (1.9–9.1)	
Alcohol consumption			
No	55.7 (53.1–58.3)	70.9 (54.8–83.0)	0.060
Yes	44.3 (41.7–46.9)	29.2 (17.0–45.3)	
Cannabis use			
No	77.8 (75.9–79.5)	84.9 (74.7–91.5)	0.151
Yes	22.3 (20.5–24.1)	15.1 (8.5–25.3)	

Data are shown as column % (95% CI), unless otherwise indicated. CI: confidence interval.

Table S2. Results of sensitivity analyses testing the association between combinations of adherence to movement behavior recommendations and substance use by gender.

	**Tobacco cigarette smoking**		**Alcohol consumption**		**Cannabis use**	
	**Boys (N= 4,431) OR (95% CI)**	**Girls (N= 5,805) OR (95% CI)**	**Boys (N= 4,431) OR (95% CI)**	**Girls (N= 5,805) OR (95% CI)**	**Boys (N= 4,431) OR (95% CI)**	**Girls (N= 5,805) OR (95% CI)**
Meeting none	1	1	1	1	1	1
PA only	0.55 (0.18–1.69)	0.74 (0.16–3.46)	**1.62 (1.05–2.48)**	1.16 (0.66–2.02)	0.90 (0.52–1.54)	0.59 (0.30–1.19)
ST only	0.72 (0.34–1.50)	0.86 (0.30–2.48)	1.35 (0.83–2.20)	0.82 (0.50–1.36)	1.14 (0.73–1.79)	0.81 (0.51–1.29)
Sleep only	0.99 (0.46–2.11)	0.55 (0.07–4.47)	**0.54 (0.33–0.87)**	0.77 (0.57–1.06)	0.90 (0.43–1.86)	0.94 (0.60–1.47)
PA & ST only	0.57 (0.19–1.66)	0.39 (0.10–1.54)	1.67 (0.93–2.99)	1.62 (0.56–4.71)	0.63 (0.34–1.18)	1.19 (0.31–4.64)
PA & sleep only	0.40 (0.08–1.99)	-[a]	1.19 (0.66–2.14)	1.53 (0.54–4.29)	0.55 (0.27–1.14)	0.36(0.06–2.25)
ST & sleep only	1.10 (0.33–3.67)	0.30 (0.07–1.40)	1.41 (0.70–2.86)	**0.49 (0.32–0.76)**	0.91 (0.46–1.79)	0.54 (0.28–1.06)
Meeting all 3	1.49 (0.49–4.49)	0.78 (0.09–6.58)	0.79 (0.51–1.22)	0.71 (0.23–2.16)	1.00 (0.42–2.40)	**0.20 (0.06–0.69)**

OR: odds ratio; CI: confidence interval; PA: physical activity; ST: screen time. Models are adjusted for age, ethnicity, subjective socioeconomic status, and body mass index z-score. OR not calculated due to empty cells.

Table S3. Results of sensitivity analyses testing the association between combinations of adherence to movement behavior recommendations and cannabis use by age group.

	**11-to-14-year-olds (N= 4,871) OR (95% CI)**	**15-to-20-year-olds (N= 5,365) OR (95% CI)**
Meeting none	1	1
PA only	0.59 (0.21–1.71)	1.09 (0.68–1.77)
ST only	0.51 (0.14–1.80)	1.04 (0.73–1.48)
Sleep only	0.48 (0.15–1.54)	1.03 (0.59–1.81)
PA & ST only	0.73 (0.24–2.24)	1.19 (0.61–2.32)
PA & sleep only	0.25 (0.03–1.89)	0.71 (0.42–1.21)
ST & sleep only	**0.61 (0.11–0.97)**	1.06 (0.64–1.75)
Meeting all 3	**0.09 (0.02–0.50)**	1.20 (0.48–2.97)

OR: odds ratio; CI: confidence interval; PA: physical activity; ST: screen time. Models are adjusted for sex, ethnicity, subjective socioeconomic status, and body mass index z-score.
